# Commentary: Accessibility of cancer drugs in Switzerland: Time from approval to pricing decision between 2009 and 2018

**DOI:** 10.3389/fpubh.2020.00517

**Published:** 2020-09-09

**Authors:** Thomas Grischott

**Affiliations:** Institute of Primary Care (IHAMZ), University and University Hospital of Zurich, Zurich, Switzerland

**Keywords:** cancer drugs, approval, reimbursement, price negotiation, health policy, public health

## Introduction

In Switzerland, medical drugs undergo a two-step procedure before they have to be reimbursed under mandatory basic health insurance: A drug must first be approved by Swissmedic, the Swiss equivalent of the US Food and Drug Administration (FDA) and the European Medicines Agency (EMA). Manufacturers may then request the approved drug to be included in the “specialities list” (SL) of the Swiss Federal Office of Public Health (FOPH). Prerequisite for SL inclusion are a favorable evaluation of the new drug's efficacy and expediency and the negotiation of a maximum price between FOPH and manufacturer. With the successful SL inclusion begins the health insurers' statutory obligation to reimburse the drug costs up to the agreed maximum price.

In their recent contribution “Accessibility of cancer drugs in Switzerland: Time from approval to pricing decision between 2009 and 2018” (1), Vokinger and Muehlematter studied the time interval between approval and SL inclusion of recent oncological drugs. A short interval is for the public benefit because most patients are not capable of paying for costly cancer drugs out of their own pockets before the drugs are included in the SL. Vokinger and Muehlematter came to the conclusion that, to the detriment of patients, the time from approval to SL inclusion increased between 2009 and 2018.

## Methods and Results

Unfortunately, the analysis presented in their article is based on partially incorrect data due to errors and inconsistencies in the original publications by the FOPH (2). For six substances, the SL inclusion dates are incorrect (Carfilzomib: 2017 instead of 2018; Pembrolizumab: 2015 instead of 2017; Dabrafenib: 2014 instead of 2017; Trastuzumab emtansin: 2014 instead of 2017; Crizotinib: 2013 instead of 2017; Abiraterone: 2012 instead of 2017). The fact that five of these misspecified inclusion years were assumed to be 2017 leads to an exaggerated peak at that particular year in figure 2 of the original article.

Given the number of corrections, it seems worth re-analyzing the data and thereby also shed some new light on its interpretation. For the following analyses, the opportunity was seized to complete SL inclusion dates until re-submission of the revised article (Niraparib: 2019; Histrelin: 2011), and to add Ruxolitinib (approved 2012, included 2014), a substance targeting hematological malignancies. Additionally, the set of substances was restricted to drugs with a time period between approval and pricing decision of no more than 3.5 years. This time window was chosen so that the reduced set includes Sonidegib with a maximum negotiation period of 1,097 days, and at the same time takes account of the fact that longer negotiations never reached a successful conclusion so far.

A naive linear regression model incorporating the remaining 64 drugs, with the approval date as independent and the time-to-pricing decision as the dependent variable, reveals a non-significant increase (*p* = 0.26) of the time-to-pricing decision of 14.4 days per year. However, this approach is inappropriate, since price negotiations for recently approved substances may be ongoing which implies that these drugs get systematically disregarded, and the 14.4 days therefore underestimate the true increase.

Presumably, it was exactly for this reason that the authors chose the SL inclusion date rather than the more intuitive approval date as independent variable for their analyses and presentation in their figure 3. However, this choice does not fully exclude all bias since, inversely to the previous situation, drugs approved before 2009 are missing among those with early inclusion dates (2009 or shortly after), thus leading to possible overestimation of the true yearly increase.

**Figure 1:**
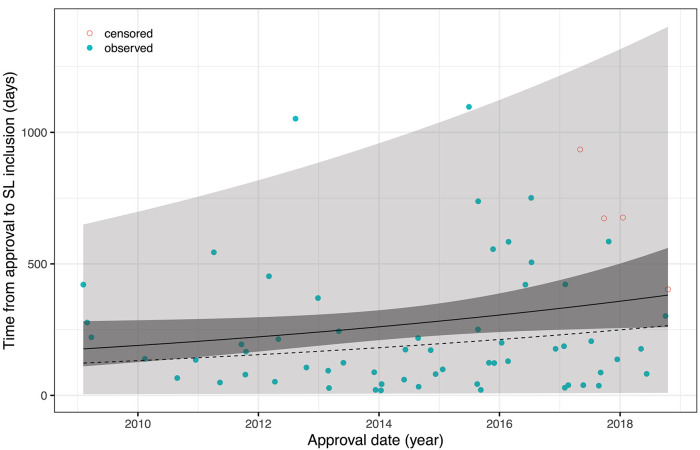
AFT model fit with 95% confidence and prediction intervals; solid line = mean, dotted line = median.

An obvious approach to account for censoring is to rely on survival analysis methodology and, in particular, to fit an accelerated failure time (AFT) model of the form log*T* = α+β·*t*+σ·ε with *T*= time-to-pricing decision (inclusion into SL), *t*= date of approval, and ε ~ *F*(ε) = 1−exp(−exp(ε)). This corresponds to *T* having a Weibull distribution with scale parameter σ and location parameter exp(α+β·*t*) in the location-scale parametrization of R's survival package which was used for calculations. The fitted model reveals a yearly increase of the time-to pricing decision by 8.24% which is not significant on the 5% level (*p* = 0.087). Figure 1 shows all data (including data censored at 25/11/2019 which is the re-submission date of the revised article, or 31/07/2019 for Olaratumab whose approval was revoked on said date before a price agreement had been reached) together with mean, median, confidence and prediction bands, and replaces figures 2, 3 of the original article (except for the rebate data).

## Discussion

Vokinger and Muehlematter found that the time-to-pricing decision has prolonged over the past 10 years. However, re-analyzing the corrected data shows that the effect is neither overwhelming in size nor significant under the chosen AFT model assumptions. Moreover, it seems that both the numbers of drugs approved as well as the duration of pricing negotiations increased discontinuously with a particularly large step in mid-2015, a singular effect which is not captured by the AFT model. It would be interesting to study whether this is in response to the evolution of cancer drug prices around that time, or due to limited personnel resources at the FOPH, more complex pricing negotiations with more frequently granted rebates, or—hypothetically—even due to the FOPH exerting pressure on the manufacturers by deliberately delaying market entry of their products while asking prices seemed too high. It would also be interesting to repeat the analysis once more data will be available.

## Author Contributions

The author confirms being the sole contributor of this work and has approved it for publication.

## Conflict of Interest

The author declares that the research was conducted in the absence of any commercial or financial relationships that could be construed as a potential conflict of interest.
